# Salivary gland carcinoma in Shanghai (2003–2012): an epidemiological study of incidence, site and pathology

**DOI:** 10.1186/s12885-019-5564-x

**Published:** 2019-04-11

**Authors:** Jin-Ye Fu, Chun-Xiao Wu, Shu-Kun Shen, Ying Zheng, Chen-Ping Zhang, Zhi-Yuan Zhang

**Affiliations:** 1 Department of Oral & Maxillofacial - Head & Neck Oncology, Ninth People’s Hospital, School of Medicine, Shanghai Jiao Tong University, Shanghai Key Laboratory of Stomatology, Shanghai, 200011 China; 2 Department of Cancer Control & Prevention, Shanghai Municipal Center for Disease Control & Prevention, Shanghai, 200336 China

**Keywords:** Oral cancer, Salivary gland neoplasms, Incidence, Epidemiology, Cancer registries, China

## Abstract

**Background:**

Salivary gland carcinoma ranks the sixth in head and neck cancers while it is relatively rare in its incidence. Epidemiological studies have been based mostly on institutional data, leading to selection bias in incidence evaluation. Most population-based cancer registries have grouped cancers of the minor salivary glands with oral cancer instead of with salivary gland carcinoma as a whole, because of the international disease coding. Thus, the incidence of salivary gland carcinoma has not been well assessed. The aim of the study is to evaluate the incidence of both minor and major salivary gland cancers in Shanghai during the years 2003–2012, and to analyse the site and histological distributions.

**Methods:**

Data from the Shanghai Cancer Registry system were extracted for patients diagnosed with malignancies of the major or minor salivary glands for the year 2003 to 2012. Pertinent socio-demographic data were obtained from the Shanghai Municipal Bureau of Public Security. The age-standardized incidence rates were calculated directly according to the world standard population. The change in incidence during the study period was analysed by comparing the rates during the first and next five years. The distributions of anatomic subsites and histology were also analysed.

**Results:**

A total of 1831 cases were identified, representing 0.35% of all malignancies during the study period. The median age was 59 and 57 years for men and women, respectively. The age-standardized incidence was 7.99 per 1,000,000 person-year, with a male-to-female ratio of 1.10. There was no significant change in the incidence during the 10-year period. The anatomic distribution confirmed the 4:1:2 rule for the parotid, submandibular, and minor glands. In men, adenocarcinoma not otherwise specified was the most common histological type followed by mucoepidermoid; in women, the mucoepidermoid was the most common histotype, followed by the adenoid cystic.

**Conclusion:**

Salivary gland carcinoma is relatively rare in incidence. However, the variations in age and sex distribution in sites and histology types suggest differences in aetiology which warrants further investigation.

## Background

Salivary gland carcinoma (SGC), estimated to be among the six most common malignancies of the head and neck [1–3], originate from the parotid, submandibular, sublingual, or from over 200 minor salivary glands that are located beneath the mucosal lining of the oral cavity and the pharynx. However, SGCs are relatively rare, estimated to represent only about 0.3% of all newly diagnosed cancers annually [4–6]. Epidemiological studies of SGC have been based mostly on institutional data, leading to selection bias in incidence evaluation. Furthermore, because of international disease coding, most population-based cancer registries have grouped cancers of the minor salivary glands with oral cancers, instead of with SGC as a whole. Thus,SGC incidence is likely underestimated.

Descriptive studies of cancer epidemiology may provide clues for aetiological research and facilitate healthcare programs. The aim of the present study was to evaluate the incidence rates and demographic characteristics as well as anatomic subsites and histology of SGC in Shanghai (population: 24 million). The study uses population-based data from the Shanghai Cancer Registry (SCR) and includes cancers of both the major and minor salivary glands.

## Methods

Incident SGC cases between 1 January, 2003 and 31 December, 2012 were identified from the Shanghai Cancer Registry system. The system is managed by the Shanghai Municipal Center for Disease Control & Preventionthat is an associate member of the International Association of Cancer Registries. The SCR is a population-based cancer registry system and has 100% coverage of Shanghai residents. Physicians are requested to report every cancer case among Shanghai residents to the SCR, at the time of diagnosis. The reporting is performed by means of a standardized cancer reporting card and is mandatory for all hospitals throughout the city. The SCR systematically collects, processes, and publishes cancer incidence data. Duplicates are consolidated in the data editing process. Death certification is used to help identify any cases missed during routine reporting.

Search criteria were created on the basis of the International Classification of Diseases for Oncology, third edition (ICD-O-3) [7], with both topography and morphology codes. The searching procedure included two parts. All cases coded as C07 and C08 in ICD-O-3 topography codes were considered as major SGCs. Cases of C00-C06 with ICD-O-3 histology codes of 8430 (mucoepidermoid carcinoma, MEC), 8200 (adenoid cystic carcinoma, ACC), 8550/8551 (acinic cell carcinoma), 8140/8190/8440/8480/8500/8570–5 (adenocarcinoma-not otherwise specified (NOS)), 8941/8940/8525/8022 (carcinoma ex-pleomorphic adenoma), 8147 (basal cell adenocarcinoma), 8310 (clear cell adenocarcinoma-NOS), and other rare types such as 8082 (lymphoepithelial carcinoma), 8982/8562 (myoepithelial carcinoma), and 8041 (small cell carcinoma), were considered as minor SGCs. Cases of other histology codes of C00-C06 were excluded. The data included demographic variables (age, sex) and clinical variables (anatomic sites and histological types). Each data has been checked by two trained clerks with the original record for the accuracy.

The corresponding population denominators were provided by the Shanghai Municipal Bureau of Public Security with age groups and sex. Ethics approval was exempted by the ethics committee of the Shanghai Ninth People’s Hospital, Medical School of Jiao Tong University, because the study was retrospective. The need for informed consent was waived, as no information to identify individual patients was included.

The incidence rates are presented as cases per 1,000,000 person-years. The age-standardized rates (ASRs) were calculated by using the direct method to the WHO World Standard Population [8]. The incidence rates were calculated according to sex, age groups, and anatomic site (parotid gland, submandibular gland, sublingual gland, minor salivary gland in palate, or other minor salivary gland).

The temporal trend of incidence was evaluated by comparing the rates of the first five and the next five years by using a Z-test for crude rates and a weighted chi-test for ASRs. The significance level was set at *P* < 0.05. Data management and all analyses were performed using SPSS 19.0 for Windows (SPSS Inc., Chicago, IL).

## Results

A total of 1831 cases (961 [52.5%] men; 870 [47.5%] women) of major and minor SGCs were retrieved from the SCR system, representing 0.35% of all malignancies in Shanghai, which were registered during the period from 2003 to 2012. The crude incidence rate was 13.26/1,000,000 person-years (men: 13.91/1,000,000; women: 12.62/1,000,000), with an ASR of 7.99/1,000,000 (men: 8.22/1,000,000; women: 7.85/1,000,000). The male to female (M/F) ratio was 1.10. The median age at the initial diagnosis of SGC was 59 years in men and 57 years in women.

The annual distribution of new cases and the corresponding incidences are showed in Table 1. Comparison of incidence rates during the first five and next five years showed that there was no significant difference between the ASRs during the study period.

**Table 1:** Yearly incidence of SGC in Shanghai from 2003 to 2012

Year	Number of cases	CR (1/1000000)	Z-test^a^	*P*-value	ASR (1/1000000)	Weighted X^2^-test^a^	P-value
2003	173	12.95	3.37	0.001	8.41	0.85	0.409
2004	169	12.56			8.61		
2005	187	13.80			8.73		
2006	155	11.37			7.18		
2007	158	11.51			6.93		
2008	187	13.51			7.96		
2009	177	12.69			7.42		
2010	227	16.07			9.18		
2011	207	14.59			8.13		
2012	191	13.43			7.89		

The incidence rates are expressed per 1,000,000 person year

*SGC* salivary gland cancer, *CR* crude rate, *ASR* age-standardized rate

^a^Comparison of the incidence rates of the first 5 years (2003–2007) and next 5 years (2008–2012)

Table 2 shows the distribution of the anatomical subsites of SGCs. In the series, over half of the SGCs occurred in the parotid gland. About one third of SGCs occurred in the minor gland, with the palate being the most common site for minor SGCs. For most sites, the median age of patients at initial diagnosis was between 55 and 59 years, while the highest median age of 73 years was observed for patients with SGCs originating from beneath the buccal mucosa. There was marked variation in the M/F ratio among incidences for anatomical subsites, ranging from 1.50 for SGC of the submandibular gland to 0.71 for minor SGCs of the palate and bucca. Except for these three sites, the M/F ratio was between 1.1 and 1.2.

**Table 2:** Incidence of SGC according to anatomic subsites in Shanghai from 2003 to 2012

Location	Males			Females			Total			Median age	M/F ratio
	No. of cases	CR	ASR	No. of cases	CR	ASR	No. of cases	CR	ASR		
Major salivary gland											
Parotid	527	7.63	4.50	449	6.51	3.99	976	7.07	4.21	57	1.17
Submandibular	150	2.17	1.24	100	1.45	0.86	250	1.81	1.04	59	1.50
Sublingual	47	0.68	0.39	40	0.58	0.35	87	0.63	0.37	58	1.18
Minor salivary gland											
Palate	77	1.12	0.68	109	1.58	1.15	186	1.35	0.92	55	0.71
Bucca	61	0.88	0.49	86	1.25	0.74	147	1.06	0.59	73	0.71
Tongue &Tongue base	32	0.46	0.28	31	0.45	0.33	63	0.46	0.30	55	1.03
Other & Unspecified	67	0.97	0.56	55	0.80	0.48	122	0.88	0.51	58	1.22
Total	961	13.91	8.22	870	12.62	7.85	1831	13.26	7.99	58	1.10

The incidence rates are expressed per 1,000,000 person-year

*SGC* salivary gland cancer; *CR* crude rate, *ASR* age-standardised rate, *M/F* male/female

Table 3 shows the distribution of the five main histological types of SGCs, which make up 55.0% of all SGCs in our series. The most common histological type in men was adenocarcinoma NOS followed by mucoepidermoid. In women, the mucoepidermoid was the most common histological type, followed by adenoid cystic. Adenocarcinoma-NOS and carcinoma ex pleomorphic adenoma were associated with a nearly 2-fold higher incidence among men than among women. Furthermore, the median ages at initial diagnosis for these two types were much higher than those of the other three types.

**Table 3:** Distribution of major histological types of SGC according to anatomical subsite in Shanghai from 2003 to 2012

	Parotid	Submandibular	Sublingual	Palate	Other minor glands	M/F ratio	Median age
Mucoepidermoid(*n* = 290)	123	19	5	92	51	0.70	50
Adenocarcinoma NOS(*n* = 228)	111	27	7	26	57	1.85	62
Adenoid cystic(*n* = 214)	42	42	36	38	56	0.83	55
Acinic cell(*n* = 147)	125	3	2	5	12	0.77	53
Carcinoma ex pleomorphic adenoma(*N* = 128)	83	23	1	13	8	1.84	60

*SGC* salivary gland cancer, *NOS* not otherwise specified, *M/F* male/female

## Discussion

Salivary gland carcinoma is relatively uncommon, leading to limitations in information regarding its epidemiological evaluations. This population-based study provides an overview of the incidence of malignancies in the major and minor salivary glands in Shanghai from the year 2003 to 2012.

In the present study, the crude incidence of SGC in Shanghai ranged annually from 11.37 to 16.07 per 1,000,000 person-years, averaging an ASR of 7.99 per 1,000,000 person-years. There was no significant difference in the ASRs of the first five and the next five years of the study period.

In contrast to other malignancies, the incidence rate of SGC has shown little variation among countries in the world. Sweden reported an SGC incidence rate of 1.32 cases per 100,000 inhabitant years during the time period between the years 1960 and 1989. The figure included both major and accessory SGCs, and showed no significant difference between the examined 10-year periods [9]. Luukkaa and his colleagues conducted a nation-wide evaluation of SGC in Finland with histological revision during the years 1991–1996 [4]. The incidence rates were in accordance with the data released by the Finland Cancer Registry, with ASRs ranging from 0.6 to 1.0/100,000 person-years during the years 1958–1998, displaying little change over the period [10]. Denmark has recently reported an ASR for SGC of 0.8/100,000/year, which is a little higher than the figure previously reported in the country [11]. The differences were probably owing to the improved standards of reporting and data-merging that improved completeness. In the Netherlands, the annual incidence of major SGC is approximately 0.7/100,000 [12]. Patrick et al. have reported annual incidence rates ranging from 0.83 to 1.38/100,000 population for both major and minor SGC during the years 1988 to 2007 in Nottingham of England [5]. There has been a modest increase in the incidence of malignant parotid neoplasms over the 20-year period in this region. According to the National Cancer Registries of England, the incidence rate for major SGC during 1990 to 2006 was between 0.7 and 0.8/100,000 population/annum. In the US, Pinkston and his group reported an incidence rate of 0.9 per 100,000 for major SGC, between 1968 and 1989 in the city of Jefferson [13], in agreement with data released by the Surveillance, Epidemiology, and End Results (SEER) program for the period spanning the years 1973 to 1992 [14]. Thus, the incidence rates among these countries have been comparable, at about 1.0/100,000 person-year.

The sex distribution of SGC in the present study suggests a higher incidence in men than that in women, with the M/F ratio of 1.10. A similar ratio has been reported in a Finland study [4], which is a little higher than that reported by studies from Sweden [9] and Denmark [11]. Guntinas-Lichius et al. described the epidemiology of head and neck cancer in Thuringia (Germany) and showed that the M/F ratio for newly diagnosed SGC was 1.64 during the period between 1996 and 2005 [2], and the incidence in men increased significantly from 0.50 to 1.57 per 100,000 persons/year over the 10-year period. Pinkston et al. reported an M/F ratio of 2.55 for SGC in the US, but the number of the cases was small [13]. Studies of the SEER database have reported that the M/F ratio for SGC was 1.15 with no change between the years 1973 and 1992 [14], while it increased to 1.29 during the period between 1992 and 2006 [1]. The male predominance in SGC incidence was once suggested to be associated with viral infection [15, 16], though this has not been confirmed by recent studies [1, 14]. In our series, the M/F ratio showed variations when analysed by anatomic subsites. SGCs of the submandibular gland showed male predominance, while those of the minor glands showed female predominance. The differences in sex distribution were consistent with those reported by Ostman [9] and Bjørndal [11]. This suggests that there are different risk factors for SGCs originating from different glands.

The median age for newly diagnosed SGC was 58 years old in our series. Women were diagnosed at an earlier age than were men, in agreement with that reported by Luukkaa [4] and Sun [14]. Other European studies have reported higher median ages at initial diagnosis, ranging from 60 to 63 years [11, 17–19], while a Brazilian study has reported an age peak for SGC incidence in the 70s [20]. In contrast, Pinkston [13] and Spiro [21] have reported a median age of 56 and 54, respectively. The aetiology of SGC at different ages-of-onset needs further investigation.

In the present study, the distribution of tumour sites was similar to that reported by other population-based studies, which revealed that over half of the SGC occurred in the parotid gland; the palate was the most common site for minor SGC; and the sublingual gland had the lowest incidence of salivary malignancies. The percentage of minor SGC in total SGCs has been reported to be 19% in Finland [4], 22.6% in Sweden [9], 24.2% in the Netherlands, and 27.9% in Denmark [11], while we reported a slightly higher incidence of 28.3%. Whether these differences are based on geographical variation, needs further evaluation.

There are some potential limitations of the study. Most importantly, the pathological diagnoses were not well-specified in 21.3% of cases. SGC presents histopathological challenges, since it displays great heterogeneity and the histological criteria are updating in the latest WHO classification. Another reason is that the SCR collects cancer reports from about 190 hospitals in Shanghai and pathological diagnosis with SGC subtyping is not mandatory for reporting. Another limitation in the study is that some SGC cases may be missed in this study. This may lead to an underestimation of the incidence. However, the reporting system to SRC is being improved in order to minimize the missing data.

## Conclusions

This population-based study on major and minor SGC incidence showed an ASR of 7.99 per 1,000,000 person-years in the period from 2003 to 2012 in Shanghai, representing 0.35% of all malignancies. The M/F ratio was 1.10 with variations among anatomic subsites. The median age at initial diagnosis was 58 years. Over half of the SGCs originated from the parotid gland. The minor SGCs accounted for 28.3% of all cases, with the palate being the most common site for minor SGC. In men, the most common histological type was adenocarcinoma NOS, followed by mucoepidermoid. The mucoepidermoid was the most common histotype in women, followed by adenoid cystic carcinoma. These epidemiological findings can help us better understand the disease and provide evidence-based clues for aetiological researches.

## Abbreviations

ACC: Adenoid cystic carcinoma
ASR: Age-standardised rate
ICD-O-3: International Classification of Diseases for Oncology, third edition
M/F: Male to female
MEC: Mucoepidermoid carcinoma
NOS: Not otherwise specified
SCR: Shanghai Cancer Registry
SEER: Surveillance, Epidemiology, and End Results
SGC: Salivary gland carcinoma
WHO: World Health Organisation

## References

[1] Boukheris H, Curtis RE, Land CE, Dores GM (2009). Incidence of carcinoma of the major salivary glands according to the WHO classification, 1992 to 2006: a population-based study in the United States. Cancer Epidemiol Biomark Prev.

[2] Guntinas-Lichius O, Wendt T, Buentzel J, Esser D, Lochner P, Mueller A (2010). Head and neck cancer in Germany: a site-specific analysis of survival of the Thuringian cancer registration database. J Cancer Res Clin Oncol.

[3] Ellis GL, Auclair PL, Gnepp DR (1991). Surgical pathology of the salivary glands. Major problems in pathology. Vol. 25.

[4] Luukkaa H, Klemi P, Leivo I, Koivunen P, Laranne J, Mäkitie A (2005). Salivary gland cancer in Finland 1991--96: an evaluation of 237 cases. Acta Otolaryngol.

[5] Bradley PJ, McGurk M (2013). Incidence of salivary gland neoplasms in a defined UK population. Br J Oral Maxillofac Surg.

[6] Parkin D, Whelan S, Ferlay J, Raymond L, Young J (1997). Cancer incidence in five continents.

[7] Fritz A, Percy C, Jack A (2000). International classification of diseases for oncology.

[8] Ahmad O, Boschi-Pinto C, Lopez AD, Murray CJL, Lozano R, Inoue M (2001). Age standardization of rates: a new WHO standard.

[9] Ostman J, Anneroth G, Gustafsson H, Tavelin B (1997). Malignant salivary gland tumours in Sweden 1960-1989--an epidemiological study. Oral Oncol.

[10] Finnish Cancer Registry (1995). Cancer incidence in Finland. Vol. 58.

[11] Bjørndal K, Krogdahl A, Therkildsen MH, Overgaard J, Johansen J, Kristensen CA (2011). Salivary gland carcinoma in Denmark 1990-2005: a national study of incidence, site and histology. Results of the Danish head and neck Cancer group (DAHANCA). Oral Oncol.

[12] Siesling S, van Dijck JA, Visser O, Coebergh JW (2003). Working Group of the Netherlands Cancer Registry. Trends in incidence of and mortality from cancer in the Netherlands in the period 1989-1998. Eur J Cancer.

[13] Pinkston JA, Cole P (1999). Incidence rates of salivary gland tumors: results from a population-based study. Otolaryngol Head Neck Surg.

[14] Sun EC, Curtis R, Melbye M, Goedert JJ (1999). Salivary gland cancer in the United States. Cancer Epidemiol Biomark Prev.

[15] Tsai CC, Chen CL, Hsu HC (1996). Expression of Epstein-Barr virus in carcinomas of major salivary glands: a strong association with lymphoepithelioma-like carcinoma. Hum Pathol.

[16] Horn-Ross PL, West DW, Brown SR (1991). Recent trends in the incidence of salivary gland cancer. Int J Epidemiol.

[17] Terhaard CH, Lubsen H, Van der Tweel I, Hilgers FJ, Eijkenboom WM, Marres HA (2004). Dutch head and neck oncology cooperative group. Salivary gland carcinoma: independent prognostic factors for locoregional control, distant metastases, and overall survival: results of the Dutch head and neck oncology cooperative group. Head Neck.

[18] Speight PM, Barrett AW (2002). Salivary gland tumours. Oral Dis.

[19] Therkildsen MH, Christensen M, Andersen LJ, Schiødt T, Hansen HS (1998). Salivary gland carcinomas--prognostic factors. Acta Oncol.

[20] Ito FA, Ito K, Vargas PA, de Almeida OP, Lopes MA (2005). Salivary gland tumors in a Brazilian population: a retrospective study of 496 cases. Int J Oral Maxillofac Surg.

[21] Spiro RH (1986). Salivary neoplasms: overview of a 35-year experience with 2,807 patients. Head Neck Surg.

